# The impact of body mass index on the pregnancy outcomes and risk of perinatal depression: Findings from a multicenter Italian study

**DOI:** 10.1192/j.eurpsy.2023.2412

**Published:** 2023-07-19

**Authors:** Antonio Ventriglio, Melania Severo, Annamaria Petito, Luigi Nappi, Salvatore Iuso, Mario Altamura, Valeria Sannicandro, Eleonora Milano, Giulia Arcidiacono, Melanie Di Salvatore, Fiammetta Gallone, Laura De Masi, Alessia Marconcini, Elisa Giannaccari, Giuseppe Maruotti, Giuseppe Luigi Palma, Mario Vicino, Antonio Perrone, Antonella Caroli, Isabella Di Pinto, Antonello Bellomo

**Affiliations:** 1 Department of Clinical and Experimental Medicine, University of Foggia, Foggia, Italy; 2 Department of Medical and Surgical Sciences, University of Foggia, Foggia, Italy; 3 Unit of Gynaecology, “Di Venere” Hospital, Bari, Italy; 4 Unit of Gynaecology, “Vito Fazzi” Hospital, Lecce, Italy; 5 Unit of Psychology, “Vito Fazzi” Hospital, Lecce, Italy; 6 Dipartimento Promozione Salute, Regione Puglia, Bari, Italy; 7 Dipartimento Promozione della Salute e del Benessere Animale, Regione Puglia, Bari, Italy

**Keywords:** body mass index, complication during pregnancy, neuroticism, obesity, perinatal depression, pregnancy, The Edinburgh Postnatal Depression Scale

## Abstract

**Background:**

Body Mass Index (BMI) is an informative factor on body fatness which has been associated to higher levels of Perinatal Depression (PD) and complications during pregnancy. We aimed to explore the impact of pre-pregnancy and postnatal BMI on the risk of Perinatal Depression and pregnancy outcomes among women recruited at their third trimester of pregnancy.

**Methods:**

We report on findings from a large multi-centre study conducted in the South of Italy and involving 1611 women accessing three urban gynaecological departments from July to November 2020. Pregnant women were assessed at their third trimester of pregnancy (T0) and after the childbirth (T1) ;The Edinburgh Postnatal Depression Scale (EPDS) has been employed for the screening of PD over time (T0 and T1) as well as other standardized measures for neuroticism, resilience, and quality of life at baseline. BMI (T0 and T1) and other socio-demographic and clinical characteristics have been collected.

**Results:**

Over-weight and obesity (higher levels of BMI) were associated with higher risk of PD (higher scores of EPDS), higher neuroticism and poorer subjective psychological well-being among enrolled women. Also, obesity and over-weight were associated with lower education, higher number of physical comorbidities, medical treatments and complications during pregnancy.

**Conclusions:**

Over-weight and obesity may impact on mental health and pregnancy outcome of women enrolled. Psycho-educational interventions aimed to improve the management of physical and emotional issues may reduce the risk of PD and complications during pregnancy.

## Introduction

Body mass index (BMI) is an anthropometric measurement for the estimation of human body fat and mass. It is an informative index derived from a person’s weight in kilograms divided by the square of height in meters. BMI screens for weight categories and indirectly may inform on health status and medical issues related to fatness [1]. Fatness is a medical and psychosocial issue and may lead to a set of metabolic and mental health issues in a bidirectional casual relationship [2, 3].

According to the National Institute of Health and World Health Organization (NIH and WHO) classifications of BMI categories, underweight is defined as a BMI under 18.5 kg/m^2^, normal-weight as BMI between 18.5 and 24.9 kg/m^2^, overweight for BMI between 25 and 29.9 kg/m^2^, and obesity for BMI greater than or equal to 30 kg/m^2^ [1].

In particular, it has been described that underweight, overweight, and obesity in pregnancy may be associated with higher maternal morbidity and show a consequent impact on pregnancy outcomes [4]. Lisonkova et al. described the distribution of BMI among 743 630 women as follows: 3.2% underweight, 47.5% normal-weight, 25.8% overweight, and 23.5% obesity. They also reported that the absolute risk (indicated as an adjusted rate-difference per 10 000 women, compared with women with normal BMI) of severe morbidity and mortality was higher among women with altered BMI compared with those reporting normal ranges of BMI: 28.8 for underweight, 17.6 for overweight and up to 61.1 for obese women [4]. More specifically, Kumpulainen et al. [5] reported that women with early overweight or obesity in pregnancy have shown higher levels of depressive symptoms and higher odds of clinically significant depressive symptoms during and after pregnancy, ranging from 23–43% and 22–36%, respectively. Also, underweight women reported 68% higher odds of clinically depressive symptoms after pregnancy [5].

Among putative mechanisms involved in the association between obesity and perinatal depression (PD), the alteration of hypothalamic–pituitary–adrenal (HPA) axis, observed in both obesity and depression, the increase of circulating glucocorticoids, higher levels of inflammation with related serum markers (including specific cytokines), oxidative stress, microbiome and psychological issues related to the changes of body-images, are widely discussed in the literature [6–8]. Pavlik and Rosculet [9], in their updated review of literature, discussed that overall evidences from studies conducted within 2020 have shown that obesity in association with its comorbidity may have a possible impact on the development of perinatal depressive symptoms: they reported findings from 5 studies confirming association between obesity and PD and 2 concluding there may be an impact of obesity on the general outcome of pregnancy but not on depressive maternal symptoms.

LaCoursiere et al. [10] reported on 1,053 pregnant women and screened depressive symptoms using the EPDS (Edinburgh Postnatal Depression Scale) [11]. Authors found that EPDS-positive scores (≥12) ranged: 40% in class 3 – obese women >32.4% in class 2 – obese women >18.8% in class 1 – obese ones >18.5% in pre-obese women >18.0% in underweight patients >14.4% in normal-weight ones; those evidences confirmed there may be an association between obesity/overweight and higher risk of depressive symptoms in pregnancy. Jani et al. [12] confirmed in their study that higher maternal early-pregnancy BMI was associated with an increased risk of developing perinatal depressive symptoms with an OR of 1.42. This evidence has been described by Dachew et al. [13] in their meta-analysis of studies regarding the role of prepregnancy BMI in the maternal depressive and anxious outcome during pregnancy and post-partum period: findings reported that prepregnancy obesity was associated with 33% higher risk of antenatal depressive symptoms whereas the association between BMI and symptoms of anxiety in the perinatal period was uncertain and not confirmed. This evidence suggested a specific exploration of depressive symptoms and their relationship with BMI scores in our sample. In addition, Santos et al. [14] in a large analysis of pregnant women cohorts from Europe, North America, and Australia, reported that higher maternal prepregnancy BMI was associated with a higher risk of complications during pregnancy (hypertension, diabetes, etc.). In fact, in an extensive review of literature by Langley- Evans et al. [15], it has been confirmed that women who report a prepregnancy BMI higher than 25 kg/m^2^ are at increased risk of complications including pre-term delivery, baby miscarriage, and stillbirth. Also, The UK Pregnancies Better Eating and Activity Trial (UPBEAT), aimed at longitudinal phenotyping of maternal antenatal depression in obese pregnant women, suggested that both depression and obesity may negatively impact on child’s adverse neurodevelopmental trajectories [16].

### Study hypothesis

In this study, we aimed to explore the impact of prepregnancy and postnatal BMI on the risk of PD and pregnancy outcomes (including complications) in a sample of 1,611 women recruited in a large observational study conducted in three gynecological departments in the south of Italy. According to the reported evidences and considering the psychological characteristics associated with higher risk of depressive symptoms in the perinatal period, we selected a set of screening tools aimed to describe the following factors: depressive symptoms with the EPDS, specifically proposed and validated for the detection of risk of PD [11, 17, 18]; the N scale of the NEO Five-Factor Inventory (NEO-FFI) describing the *neuroticism* as a personality trait associated with higher risk of mood disorders [5, 19]; The Experience in Close Relationship (ECR) detecting the attachment-related *anxiety* and *avoidance* in close relationships, both predisposing to develop affective symptoms during specific transition stages such as pregnancy [20, 21]; The Connor–Davidson Resilience Scale (CD-RISC) [22] exploring the personal resilience abilities to withstand adversity or stressful experiences such as pregnancy and its challenges; and The WHOQOL BREF measuring the quality of life (QOL) as associated factor of depression [23, 24]. These characteristics have been also explored through the different BMI classes to test their associations with underweight, overweight, and obesity.

## Methods and materials

### Sample and study design

This study has been conducted in three large urban gynecological departments of Puglia, a region in the south of Italy: Foggia (Policlinico Riuniti di Foggia), Bari (Ospedale Di Venere) Lecce (Ospedale Vito Fazzi). It is part of a screening/prevention program aimed to detect depressive symptoms and associated risk factors promoted by the regional agency for health prevention. 1664 women have been included, all consecutively admitted from July to November 2020, during their third trimester of pregnancy and followed-up during the *peripartum*, even after childbirth: they were all recruited except by exclusion criteria which included women reporting an intellectual disability, poor language proficiency, age < 18 years old or those refusing to provide informed consent with their partners. The assessment has been based on standardized and validated psychological tools for detecting personality traits, depressive symptoms, as well as other psychological characteristics (described below). Also, BMI, anthropometric measures, sociodemographics, and pregnancy characteristics have been collected. Assessments have been performed at the enrolment (T0: 15–45 days before the delivery) and within the seventh day after childbirth (T1). Assessment at T0 collected all psychological characteristics related to women’s personality traits, sociodemographics, as well as current symptoms of depression and retrospective information regarding the BMI, recorded at the beginning of the pregnancy: beyond the baseline measurements, we decided to set the detection of depressive symptoms before the delivery according to evidences and suggestions from the international literature [11]. Similarly, the variation of scores for the risk of depression has been evaluated after childbirth (T1) since the delivery may represent a stressful event from a psychological and psychical point of view as well as a trigger for depressive symptoms; also, BMI may be variable during the pregnancy and the amplitude of variation from baseline is higher after the childbirth [5]. All women and their partners have provided their written informed consent with an agreement on privacy and anonymous data-processing. The investigation has been conducted by well-trained psychologists, psychiatrists, psychiatric trainees, and one statistician, and data were based on a multicenter collection (cities of Foggia, Bari, and Lecce).

### Assessment tools

The detection of depressive symptoms and relative risk of PD has been conducted with the EPDS [11]. EPDS scores as well as other psychological characteristics, sociodemographic variables, and information on personal medical history and pregnancy, including anthropometric measures, have been all collected at T0 and T1 evaluations.

The EPDS [11] is widely employed and recognized as a validated and standardized instrument for the screening of PD in different socio-cultural settings. It collects depressive symptoms within the last seven days of observation and includes 10 items based on a 4-point Likert scale with a total score ranging from 0 to 30. As suggested by the international literature, we considered a total score ≥ 12 as significantly associated with a clinical risk of PD (according to the National Institute for Health and Care Excellence guidelines, NICE [17]). In this research protocol, the Italian version by Benvenuti et al. has been employed [18].

The NEO-FFI by McCrae and Costa [19] has been employed for detecting neuroticism among pregnant women: this tool includes 60 items with three subscales on Neuroticism (N), Extraversion (E), and Openness (O). We considered scores from the N subscale based on 12 items ranging on a 5-point Likert scale and describing Neuroticism, defined as a fundamental personality trait associated with higher levels of anxious-depressive or negative experiences (e.g., worry, loneliness, fear, frustration, anger, etc.).

The ECR by Brennan [20] has been also administered for testing differences regarding attachment-related Anxiety and Avoidance in close relationships. It is a self-administered tool based on 36 items describing the individual attachment style to the partner as well as assessing anxiety or avoidance. The Italian version by Picardi et al. has been employed [21].

The CD-RISC [22] explores resilience as the ability to withstand adversity and bounce back from difficult life events. It is based on 25 items exploring some resilience domains: personal competence, high standards, and tenacity; trust in one’s instincts, tolerance of negative affect, and strengthening effects of stress; positive acceptance of change and secure relationships; control; spiritual influences. Higher scores indicate a higher level of personal resilience.

The WHOQOL BREF measures QOL and is a shorter version of WHOQOL-100, an assessment tool proposed by the World Health Organization – WHOQOL Group in 1996 [23]. It explores the subjective QOL in the context of the culture and values system, in relation to personal goals, expectations, standards, and concerns. It is based on 26 items focused on the following domains: physical health; psychological health; social relationships; environment. The Italian version by de Girolamo et al. [24] has been employed.

### Ethical approval

This study is part of a large program designed and conducted by the Unit of Psychiatry at the University of Foggia in cooperation with the Units of Gynecology at Policlinico Riuniti di Foggia/University of Foggia (Foggia, Italy), Ospedale Vito Fazzi di Lecce (Lecce, Italy) and Ospedale Di Venere di Bari (Bari, Italy). Ethical approval has been provided by the Regione Puglia with two specific deliberations “DGR n. 1392 released on 2 August 2018 *and* DGR n. 2294 released on 11 December 2018”. This project has been also promoted by the Department of Health Promotion of the Regione Puglia entitled “*Governo dell’assistenza alle persone in condizione di fragilità”* and approved with a specific deliberation n. 65 released on 12 March 2019.

Local approvals have been also obtained by the following committees: Policlinico Riuniti di Foggia/University of Foggia (Foggia, Italy), Ospedale Vito Fazzi di Lecce (Lecce, Italy) and Ospedale Di Venere di Bari (Bari, Italy). All participants provided written informed consent and agreement on privacy and data-managing; participation was free of any charge. Findings, data collected, and any information were treated with confidentiality, equality, and justice, respecting the Helsinki principles.

### Statistical analyses

Statistical analyses employed commercial microcomputer programs (Statview, SAS Corp., Cary, NC; Stata, Stata Corp., College Station, TX). Data have been presented as means ± standard deviations (SDs), percentages (%), or 95% confidence intervals (CIs). Continuous data were compared by analysis of variance (ANOVA) methods (*F*), and categorical data by contingency tables (χ*^2^*); Bonferroni correction was used to correct for multitesting; associations of specific measures of observation were tested by non-parametric Spearman rank correlation (*r*). Multivariate logistic regression modeling of association of selected factors to BMI [yielding odds ratios (OR) and their 95% confidence intervals (CIs)], by stepwise inclusion of factors in order of their strength (*p-*value) of preliminary bivariate association with BMI was carried out. Findings were considered statistically significant with two-tailed *p* ≤ 0.05.

## Results

### Sample characteristics and assessments at T0 and T1

1,611 women, aged 32.4 ± 5.50 years old, were included in the program during their third trimester of pregnancy from July to November 2020. Approximately 79% of women accessing the three Units of Gynecology agreed to join the study.

The following variables have been described in the whole sample (Table 1): 50.2% of women (*n =* 843) reported post-secondary education level as well as 38.5% (*n =* 640) a higher-education; 66.3% of women were married (*n =* 1104); employment has been reported in 55.9% of participants (*n =* 924); 2.40% of them (*n =* 40) reported a previous mood disorder in the 6 months preceding the pregnancy, 1.32% (*n =* 22) an eating disorder; 10.5% (*n =* 176) an anxiety disorder; 28% (*n =* 473) reported a previous spontaneous abortion, 50.9% (*n =* 826) a premenstrual syndrome.

**Table 1.** Sample characteristics and assessments at T0 and T1 (*N =* 1,611)

Characteristics	T0	T1
	mean ± s.d. *or* %(*n*)	
Current age (years old)	32.4 ± 5.50	
Education (high school)	50.2% (*n =* 843)	
Marital status (married)	66.3% (*n =* 1,104)	
Employment (yes)	55.9% (*n =* 924)	
Mental disorder(s) (yes, diagnosed in the previous 6 months)	14.2% (*n =* 238)	
Previous abortion(s) (yes)	28% (*n =* 473)	
Premenstrual syndrome (yes)	50.9% (*n =* 826)	
Complications during pregnancy (yes)	29.7% (*n =* 445)	
Medical comorbidities (yes)	24.2% (*n =* 391)	
Body mass index		
Underweight	7.20% (*n =* 116)	0.06% (*n =* 1)
Normal-weight	61.5% (*n =* 992)	25.8% (*n =* 416)
Overweight	20.6% (*n =* 333)	58.1% (*n =* 937)
Obesity	10.5% (*n =* 170)	15.9% (*n =* 257)
EPDS ≥ 12	14.5% (*n =* 235)	8.13% (*n =* 131)
NEO	14.7 ± 7.31	
ECR-S anxiety	41.9 ± 18.7	
Avoidance	29.9 ± 13.6	
CD-RISC	78.2 ± 13.6	
WHOQOL psychological well-being	12.3 ± 2.48	
Social relationships	12.1 ± 1.83	

Abbreviations: BMI, body mass index, kg/m^2^; CD–RISC, The Connor–Davidson resilience scale; ECR-S, The experience in close relationship scale; EPDS, The Edinburgh Postnatal Depression Scale; NEO, The N scale of the 60 items NEO Five-Factor Inventory; s.d., standard deviation; The WHOQOL BREF, World Health Organization Quality of Life.

Complications during pregnancy ranged: gestational diabetes 145 (8.75%) > blood loss 72 (4.34%) > hypertension 14 (0.84%) > leakage of amniotic fluid 6 (0.36%); other complications rated 6.27%, ≥2 complications 3.55%, none 70.3% (*n =* 1166), respectively. Medical comorbidities (*n =* 391; 24.2%) mostly included diabetes, hypertension, and thyroiditis with related specific medical treatments.

The *prepregnancy* body mass index (BMI T0), as reported by participants rated: 7.20% (*n =* 116) underweight women (BMI < 18.5); 61.5% (*n =* 992) normal-weight (BMI: 18.5–24.9); 20.6% (*n =* 333) overweight; 10.5% (*n =* 170) obese women (BMI ≥ 30). BMI was remeasured at T1 with the following results: 0.06% (*n =* 1) underweight; 25.8% (*n =* 416) normal-weight; 58.1% (*n =* 937) overweight; 15.9% (*n =* 257) obese women; total mean BMI variation during the follow-up (Δ BMI T1–T0) was (+) 3.45 ± 2.20 (Table 1).

The EPDS general score in the sample was 6.46 ± 4.49, reporting a general level of depressive symptoms below the significant cutoff: 14.5% (*n =* 235) of women reported a significant level of depressive symptoms (EPDS≥12) with a high risk of PD at T0 whereas 8.13% (*n =* 131) at T1 with a mean score 14.6 ± 2.95. Neuroticism, as measured with the NEO Five-Factor Inventory and considered as an associated risk factor for PD, scored 14.7 ± 7.31 (low general level of neuroticism; not shown); Anxiety and Avoidance in Close Relationships assessed by ECR-S scored 41.9 ± 18.7 and 29.9 ± 13.6, respectively, confirming low levels (Table 1). CD-RISC assessment for personal resilience, as a personal protective factor for PD, has shown a total score of 78.2 ± 13.6, with a medium–low level of resilience (not shown). Finally, the QOL of participants has been evaluated at WHOQOL BREF reporting total sub-scores of 12.3 ± 2.48 and 12.1 ± 1.83, describing psychological well-being and quality of social relationships in the normal range.

Of 1611, *n =* 1541 were retested within 7 days after delivery (T1) using EPDS. Of these, 8.13% (*n =* 131) reported a significant level of depressive symptoms with a relevant risk of PD (EPDS≥12).

### Sample characteristics and BMI categories

Table 2 shows a bivariate analysis of sample characteristics at baseline and their description across different BMI categories, as considered in the study.

**Table 2.** Sample psychological characteristics and BMI at baseline (T0) and repeated measure (EPDS) at T1

	Underweight	Normal-weight	Overweight	Obese	*F* or *χ*^2^	*p*
	(BMI: <18.5)	(BMI: 18.5–24.9)	(BMI: 25.0–29.9)	(BMI: ≥30.0)		
	*n =* 116	*n =* 992	*n =* 333	*n =* 170		
	mean ± s.d.	mean ± s.d.	mean ± s.d.	mean ± s.d.		
EPDS T0	6.45 ± 4.22	6.44 ± 4.41	6.54 ± 4.53	6.75 ± 4.78	0.252	0.859
EPDS, T1	5.16 ± 3.40	5.44 ± 3.89	5.83 ± 4.30	6.20 ± 4.30	2.385	0.067
EPDS T0 ≥ 12	14 (12.0)	139 (14.0)	51 (15.3)	24 (14.1)	17.5	**0.025**
EPDS T1 ≥ 12	6 (5.17)	68 (6.85)	35 (10.5)	19 (11.1)	24.5	**0.002**
NEO	13.6 ± 7.60	14.3 ± 7.01	15.4 ± 7.71	16.1 ± 7.33	4.760	**0.002**
ECR-S anxiety	38.0 ± 16.6	42.0 ± 18.6	42.4 ± 18.8	42.9 ± 18.8	1.971	0.116
Avoidance	28.1 ± 11.9	29.9 ± 13.6	30.2 ± 13.6	30.8 ± 14.4	0.992	0.395
CD-RISC	77.5 ± 13.7	78.3 ± 13.1	77.7 ± 13.7	78.0 ± 13.4	0.250	0.861
WHOQOL psychological well-being	12.8 ± 2.62	12.4 ± 2.41	11.9 ± 2.53	12.0 ± 2.52	5.431	**0.001**
Social relationships	12.4 ± 1.67	12.1 ± 1.77	12.0 ± 1.97	11.9 ± 2.00	1.547	0.200

Abbreviations: BMI, body mass index, kg/m^2^; CD–RISC, The Connor–Davidson resilience scale; ECR-S, The experience in close relationship scale; EPDS, The Edinburgh Postnatal Depression Scale; NEO, The N scale of the 60 items NEO Five-Factor Inventory; s.d., standard deviation; The WHOQOL BREF, World Health Organization Quality of Life.

Bold: statistically significant

Even the EPDS scores did not significantly vary across BMI prepregnancy categories, levels of depressive symptoms and related risk of PD was significantly higher among overweight and obese women at baseline (T0): in particular 15.3% (*n =* 51) of overweight women and 14.1% (*n =* 24) of obese women reported EPDS levels≥12 (*p =* 0.025). At T1 retest, overweight and obese women reported higher levels of PD risk with EPDS-positive cases of 11.1% (*n =* 19) and 10.5% (*n =* 35) respectively (*p =* 0.002). Also, levels of Neuroticism (NEO), as risk factor for PD, were higher among overweight and obese women than normal and under-weigh ones (*p =* 0.002). These findings confirm evidences from the literature recognizing neuroticism as a specific risk factor for PD [25–28] as well as a psychological *endophenotype* of affective disorders (as further discussed in this manuscript) [29]. Consequently, levels of psychological well-being (as measured at WHOQOL scale) were significantly much higher among underweight and normal-weight women (scores>12) whereas overweight and obese pregnant women scored ≤12 (*p =* 0.001). No significant differences across BMI categories regarding the QOL related to social relationships were found.

Among the sociodemographic and clinical characteristics of the sample (Table 3), we found that underweight women were significantly younger than in other groups: underweight, 30.8 ± 6.75 < overweight, 32.0 ± 5.53 < obese, 32.6 ± 5.27 < normal-weight, 32.8 ± 5.32 (*p =* 0.0014). Women were all more likely to be married in all groups (*p =* 0.0004), mostly employed, whereas overweight and obese women reported a significantly higher level of education (<0.0001).

**Table 3.** Sociodemographic and clinical characteristics and BMI at baseline (T0)

	Underweight	Normal-weight	Overweight	Obese	*F* or *χ*^2^	*p*
	(BMI: <18.5)	(BMI: 18.5–24.9)	(BMI: 25.0–29.9)	(BMI: ≥30.0)		
	*n =* 116	*n =* 992	*n =* 333	*n =* 170		
	mean ± s.d./*n* (%)	mean ± s.d./*n* (%)	mean ± s.d./*n* (%)	mean ± s.d./*n* (%)		
Age (years old)	30.8 ± 6.75	32.8 ± 5.32	32.0 ± 5.53	32.6 ± 5.27	5.220	**0.0014**
Marital status					40.18	**0.0004**
Married	50 (43.1)	673 (67.8)	227 (68.1)	120 (70.5)		
Single	3 (2.58)	11 (1.10)	3 (0.90)	1 (0.58)		
Other	63 (54.3)	308 (31.0)	103 (31.0)	49 (28.9)		
Employment					13.23	**0.0042**
Yes	66 (56.8)	582 (58.6)	157 (47.1)	93 (54.7)		
No	50 (43.2)	410 (41.4)	176 (52.9)	77 (45.3)		
Education					56.04	**<0.0001**
High school	53 (45.6)	468 (47.1)	191 (57.3)	96 (56.4)		
University	45 (38.7)	438 (44.1)	91 (27.3)	45 (26.4)		
Other	18 (15.7)	86 (8.80)	51 (15.4)	29 (17.2)		
Previous mental disorder					15.77	0.2020
No	96 (82.7)	823 (82.9)	277 (83.1)	136 (80.0)		
Yes, depression	4 (3.44)	39 (3.93)	20 (6.00)	9 (5.29)		
Yes, other	16 (13.8)	130 (13.1)	36 (10.9)	25 (14.7)		
Previous psychopharmacotherapy					3.030	0.3870
Yes	1 (0.90)	27 (2.72)	5 (1.50)	3 (1.76)		
No	115 (99.1)	965 (97.2)	328 (98.5)	167 (98.2)		
Previous psychotherapy					3.320	0.3448
Yes	3 (2.6)	46 (4.7)	13 (4.00)	6 (3.60)		
No	113 (97.4)	946 (95.3)	320 (96.0)	164 (96.4)		
Family history of mental illness					33.72	0.0136
Yes	28 (24.2)	255 (25.8)	74 (22.3)	35 (20.5)		
No	88 (75.8)	737 (74.2)	259 (77.7)	135 (79.5)		
Tobacco use					2.429	0.4883
Yes	4 (3.50)	42 (4.30)	20 (6.10)	7 (4.20)		
No	112 (96.5)	950 (95.7)	313 (93.9)	163 (95.8)		
Coffee and energy-drinks use					4.006	0.2609
Yes	66 (56.9)	535 (54.0)	196 (58.9)	86 (50.6)		
No	50 (43.1)	457 (46.0)	137 (41.1)	84 (49.4)		
Alcohol use					2.588	0.4596
Yes	1 (0.90)	4 (0.50)	3 (1.00)	1 (0.60)		
No	115 (99.1)	988 (99.5)	330 (99.0)	169 (99.4)		
Physical comorbidity					32.69	**<0.0001**
Yes	29 (25.0)	213 (21.5)	78 (23.5)	71 (41.8)		
No	87 (75.0)	779 (78.5)	255 (76.5)	99 (58.2)		
Medical treatments					18.32	**0.0004**
Yes	19 (16.4)	174 (17.6)	61 (18.4)	53 (31.2)		
No	97 (83.6)	818 (82.4)	272 (81.6)	117 (68.8)		
Dysmenorrhea					1.098	0.7776
Yes	52 (44.9)	455 (45.9)	143 (43.0)	80 (47.1)		
No	64 (55.1)	537 (54.1)	190 (57.0)	90 (52.9)		
Premenstrual syndrome					0.270	0.9656
Yes	60 (51.8)	517 (52.2)	178 (53.5)	86 (50.6)		
No	56 (48.2)	475 (47.8)	155 (46.5)	84 (49.4)		
Previous abortions					9.080	0.1691
Yes	23	279	102	58		
No	93 (80.1)	713 (71.8)	231 (69.3)	112 (65.8)		
Previous voluntary termination of pregnancy					3.036	0.3862
Yes	9 (7.80)	96 (9.70)	28 (8.50)	21 (12.4)		
No	107 (92.2)	896 (90.3)	305 (91.5)	149 (87.6)		
Previous mental disorders in pregnancy					10.53	0.5689
Yes	15 (13.0)	145 (14.7)	48 (14.5)	17 (10.0)		
No	101 (87.0)	847 (85.3)	285 (85.5)	153 (90.0)		
Complications of current pregnancy					97.81	**<0.0001**
Yes	28 (24.2)	275 (27.8)	105 (31.6)	74 (43.6)		
No	88 (75.8)	717 (72.2)	228 (68.4)	96 (56.4)		
Hyperemesis gravidarum					3.111	0.3748
Yes	56 (48.3)	519 (52.4)	189 (56.8)	90 (53.0)		
No	60 (51.7)	473 (47.6)	144 (43.2)	80 (47.0)		
Nausea					7.443	0.0591
Yes	76 (65.6)	750 (75.7)	236 (70.9)	125 (73.6)		
No	40 (34.4)	242 (24.3)	97 (29.1)	45 (26.4)		

Abbreviations: BMI, body mass index, kg/m^2^; s.d., standard deviation.

Bold: statistically significant

In addition, as expected, obese and overweight women reported significantly higher number of complications during pregnancy as follows (Table 3): obese, 43% > overweight, 31% > normal-weight, 27% > underweight, 24% (*p* < 0.0001). Consistently, obese women reported more physical comorbidities and ongoing medical treatments: medical comorbidity (mostly diabetes, hypertension, and thyroiditis) rated 41.8% among obese >25% underweight > 23.5% overweight > 21.5% normal-weight (*p* < 0.0001); related medical treatments rated 31.2% among obese >18.4% overweight > 17.6% normal-weight > 16.4% underweight (*p =* 0.0004).

In a simple regression model, we found that factors such as depressive symptoms at T1 (EPDS scores) and Neuroticism increased with BMI at baseline (T0) whereas, consistently, psychological well-being (WHOQOL) decreased when BMI T0 was higher (*all p* < 0.0087; Table 4). After childbirth (T1), the increase in BMI (T1) was significantly associated with the increase in depressive symptoms (EPDS T1), Neuroticism, anxiety, and avoidance (ECR-S), whereas levels of QOL (psychological well-being and social relationships at WHOQOL) decreased when BMI T1 was higher (*all p* < 0.0254; Table 4). In addition, as discussed before, complications during pregnancy, as well as physical comorbidities, were associated with higher ranges of BMI variation over time (ΔBMI: BMI T1- BMI T0; Table 5).

**Table 4.** Psychological baseline characteristics and BMI at T0 and T1

	BMI T0 (*r)*	*p*	BMI T1 (*r)*	*p*
EPDS T0	+0.019	0.4501	+0.39	0.1495
EPDS, T1	+0.076	**0.0087**	+0.063	**0.0127**
NEO	+0.055	**0.0003**	+0.168	**0.0001**
ECR-S	+0.011	0.0597	+0.335	**0.0030**
Anxiety				
Avoidance	+0.012	0.1296	+0.199	**0.0163**
CD-RISC	−0.002	0.8496	−0.004	0.9652
WHOQOL psychological well-being	−0.167	**0.0002**	−0.057	**0.0001**
Social relationships	−0.091	0.1317	−0.25	**0.0254**

Abbreviations: BMI, body mass index, kg/m^2^; CD–RISC, The Connor–Davidson resilience scale; ECR-S, The experience in close relationship scale; EPDS, The Edinburgh Postnatal Depression Scale; NEO, The N scale of the 60 items NEO Five-Factor Inventory; s.d., standard deviation; The WHOQOL BREF, World Health Organization Quality of Life.

Bold: statistically significant

**Table 5.** Sample baseline characteristics and BMI variation (Δ BMI: BMI T1–BMI T0) overtime

	Δ BMI (BMI T1–BMI T0)	*F* or *r*	*p*
EPDS T0	–	0.014	0.2499
EPDS, T1	–	−0.032	0.4994
NEO	–	−0.017	0.8324
ECR-S anxiety	–	+0.357	0.0894
Avoidance	–	+0.215	0.1628
CD-RISC	–	+0.047	0.7622
WHOQOL psychological well-being	–	+0.012	0.6806
Social relationships	–	−0.023	0.2693
Age (Years old)	–	−0.134	**0.0311**
Substance abuse (all)		1.315	0.2516
No	3.46 ± 2.20		
Yes	2.00 ± 1.02		
Physical comorbidity		9.116	**0.0026**
No	3.16 ± 2.16		
Yes	3.55 ± 2.21		
Complications of current pregnancy		4.758	**<0.0001**
No	2.27 ± 2.71		
Yes	3.58 ± 2.22		
Medical treatments		2.328	0.1273
Yes	3.28 ± 2.18		
No	3.49 ± 2.21		
Hyperemesis gravidarum		0.141	0.7070
No	3.47 ± 2.06		
Yes	3.43 ± 2.33		
Nausea		3.076	0.0796
No	3.51 ± 2.23		
Yes	3.29 ± 2.13		

Abbreviations: BMI, body mass index, kg/m^2^; CD–RISC, The Connor–Davidson resilience scale; ECR-S, The experience in close relationship scale; EPDS, The Edinburgh Postnatal Depression Scale; NEO, The N scale of the 60 items NEO Five-Factor Inventory; s.d., standard deviation; The WHOQOL BREF, World Health Organization Quality of Life.

Bold: statistically significant

We considered those factors preliminarily associated with BMI categories in the bivariate analyses (Tables 2 and 3) for subsequent logistic multivariate modeling. Characteristics that remained significantly and independently associated were (in descending order of statistical significance): a) more complications during pregnancy in obese women; b) more complications during pregnancy in overweight women; c) lower level of education in overweight women; d) more Neuroticism (NEO) in obese women (Table 6).

**Table 6.** Multivariate logistic regression model of factors associated with Body Mass Index (underweight, overweight and obesity) at baseline (T0)

	OR (95%CI)	*χ*^2^	*p*
More complications during pregnancy in obese women	0.34(0.06–2.06)	13.4	0.0001
More complications during pregnancy in overweight women	0.71(0.13–3.7)	9.59	0.002
Lower level of education in overweight women	2.45(1.47–4.08)	11.9	0.008
More Neuroticism (NEO) in obese women	2.71(0.12–59.2)	7.14	0.049

Abbreviations: NEO, The N scale of the 60 items NEO Five-Factor Inventory; OR, odds ratio.

## Discussion and conclusions

This study aims to test the role of BMI in the screening and prediction of risk for PD during pregnancy. Preliminary analyses have shown that the risk of depression (EPDS≥12) was higher among overweight and obese women ranging from 14.1–15.3%. This evidence is in line with the international literature [5, 10] even if the percentage of prevalence of PD risk seems to be lower than in other reports, ranging from 18–40% among overweight and obese groups. This may reflect that the larger range of prevalence reported in the literature includes different findings analyzed in systematic reviews or metanalyses as well as different cutoffs and categories employed by the authors for the definition of risk of depression at EPDS and BMI variations. Despite these differences, the association between higher BMI and increased scoring at EPDS is clearly confirmed in this report, also by the analysis of simple regression as shown in Table 3. In particular, the PD risk, as measured by EPDS≥12, at T1 increased with both BMI measurements at baseline T0 (*p =* 0.0087) and T1 (*p =* 0.0127). This adds to the evidence that obesity and its associated comorbidity may have an impact on the development of perinatal depressive symptoms. As discussed, the HPA axis deregulation, involved in both obesity and depression, may be a biological key factor for explaining the increase of circulating glucocorticoids and inflammation markers leading to higher levels of physical comorbidity and psychopathological symptoms [6–8]. The role of other personality and psychological characteristics involved in the vulnerability panel of depression and obesity were also considered and here discussed. The EPDS scores among overweight and obese women in our sample ranged between 14.1–15.3 at T0 and 10.5–1.1 at T1, showing lower levels of depressive symptoms than those reported by other authors [10]. However, the odds ratio for depressive symptoms among overweight/obese women at T1 in our sample was 1.56 (95% CI: 1.0785 to 2.2742; z = 2.357; *p* = 0.0184) and confirmed the evidence by Jani et al. reporting an OR = 1.42 [12].

An interesting finding indicated that Neuroticism is a key factor significantly involved in the PD-risk assessment and it has shown differences among BMI categories in the bivariate as well as multivariate analysis. In fact, obese and overweight pregnant women at baseline (BMI T0; Table 2) reported a higher level of neuroticism, as measured with the N scale of NEO: overweight/obese women, 15.4–16.1 *versus* underweight/normal-weight, 13.6–14.3. This evidence has been confirmed in the multivariate logistic regression modeling where obese women reported significantly higher levels of neuroticism with an OR = 2.71 (*p =* 0.049). In a previous preliminary descriptive study, Bellomo et al. [25] confirmed that higher levels of neuroticism were detected among women reporting EPDS scoring ≥12 with a significant positive correlation between NEO scores and depressive symptoms. Authors argued that neuroticism is largely recognized as a specific risk factor for PD by the international literature [26–28]; also it has been proposed as a psychological *endophenotype* of affective disorders [29] as well as a personality characteristic leading to adjunctive vulnerability to personal stress in the PD pathogenesis [29, 30]. In this study the positive correlation between Neuroticism and BMI has been confirmed both at baseline (T0) and T1 (*r* = +0.055, *p =* 0.0003; *r* = +0.168, *p =* 0.0001). Recently, Chen et al. [31] used a structural equation modeling to explore the pathways from neuroticism and other factors to PD among 773 women in the third trimester of pregnancy. Depression was positively correlated with neuroticism and negatively with social support and sleep quality, confirming the evidence that neuroticism may have a direct effect on the risk of depression and should be routinely assessed among pregnant women [31, 25]. Moreover, Shakeri et al. [32] reported findings from a longitudinal study on the relationship between a mother’s personality traits, eating behaviors, and maternal weight gain during pregnancy. They concluded that high neuroticism was significantly associated with higher levels of consumption of energetic food directly related to weight gain. Moreover, Sutin and Terracciano [33] specifically explored the relationship between neuroticism and BMI on a large sample of 5,150 subjects from the general population (50% of them were females) and described that high levels of Neuroticism were associated with higher BMI and risk for obesity, with the behavioral factors, including attitudes to physical activity, diet, regular meal rhythms, as characteristics directly impacted by Neuroticism and accounting for the 50% of the association between Neuroticism and BMI. We argue that Neuroticism may be involved in the vulnerability panel of depression and eating behaviors with a possible role of mediating factor between depression and obesity. Ad-hoc studies might be of interest in order to explore this suggestive evidence from the literature findings.

Psychological well-being significantly differed across BMI categories in our bivariate analysis. In fact, obese and overweight women reported slightly lower scores at WHOQOL psychological well-being subscale than normal- and underweight patients (*p =* 0.001). Probably this may reflect the evidence that higher levels of neuroticism, depressive symptoms as well as more physical comorbidities and complications (as further discussed), impact on general QOL and well-being. The relationship between overweight/obesity and QOL has been also tested in a simple regression model with an inverse correlation between BMI and all WHOQOL scores at T1 (psychological well-being, *r =* −0.057, *p =* 0.0001; social relationships, *r* **=** −0.25, *p =* 0.0254) and well-being and prepregnancy BMI (psychological well-being, *r* = −0.167, *p =* 0.0002). Interestingly, this finding may be supported by the significant increase of anxiety and avoidance in close relationships founded in the regression model showing a positive correlation between BMI increase at T1 and scores recorded at ECR-S (anxiety, *r* = +0.335, *p =* 0.0030; avoidance, *r* **=** +0.199, *p =* 0.0163). Both anxiety and avoidance in the experience of close relationships are consistently expected with higher levels of depressive symptoms and neuroticism after childbirth and reasonably impact on quality of relationships and psychological well-being. Since ECR-S variables did not show a significant correlation at baseline, they did not impact on WHOQOL social relationships score at T0.

Clinical and sociodemographic characteristics significantly associated with BMI categories were: education, physical comorbidities, and complications during pregnancy (*all p* < 0.0001); marital status and medical treatments (*all p =* 0.0004); age (*p =* 0.0014) and employment (*p =* 0.0042). It may be expected that higher weight gain is associated with more physical comorbidities and complications during pregnancy. Recent evidences reported that higher maternal prepregnancy BMI and gestational weight gain are associated with higher risk of gestational hypertension, diabetes and baby size issues at birth [11]. In fact, in our sample major comorbidities included gestational hypertension and diabetes. Moreover, Santos et al. [11] confirmed that high gestational weight gain was associated with the highest risk of any pregnancy complication with OR: 2.51 (95% CI 2.31–2.74) and 23.9% of any pregnancy complication attributable to maternal overweight/obesity. Similarly, Langley-Evans et al. [13] reported that all pregnancy complications were more likely with overweight, obesity, and gestational weight gain. We also confirmed this last evidence since complications as well as physical comorbidities, even slightly, were both positively correlated to the BMI (Δ T1–T0) variation over time (*r* = 4.758, *p* < 0.0001; and *r* = 9.116 *p =* 0.0026, respectively; Table 5). Consequently, complications during pregnancy were confirmed as strongly associated with obesity and overweight in the multivariate logistic regression modeling with OR ranging from 0.30–0.71 (*all* p ≤ 0.002).

Surprisingly, education was a sociodemographic factor significantly associated with BMI in the bivariate as well as multivariate analyses. In particular, overweight and obese women reported lower level of education (considering combined frequencies of high school-level and university-level across the BMI categories: underweight 84% of upper education, normal-weight 91%, overweight 81%, obesity 82%). Suggestively, a lower level of education may have a role in both depressive and weight-gain outcomes. For instance, Kim et al. [34] reported that the mean score of depression measured with the Patient Health Questionnaire- 9 (PHQ-9) [35] during pregnancy was associated with low education level as well as decreased with subjective health status (*all p* < 0.001). Conversely, Banite et al. [36] have shown that employed women presented a lower level of PD whereas a higher level of education was positively associated with a higher risk of depression. In our sample, similarly, the rate of employment was slightly higher among normal-weight women than overweight and obese ones.

### Strengths and limitations

Limitations of this study may include the lack of further information regarding other variables and factors potentially impacting on the risk of depression and obesity. The assessment and measurements were performed at baseline and after childbirth only, a longer follow-up might have been more informative. Also, the prepregnancy BMI was declared by the participants on the base of gynecologic registers: there may be a methodological weakness in reporting BMI scores instead of measuring them. In addition, of 1,611 women enrolled at T0, 1541 were retested at T1 with a total (−) 4.34% of drop out. However, the amount of collected variables was large and factors were considered as suggested by the evidences of the international literature. Also, the measurement of baseline BMI (T0) was recorded by highly qualified personnel in a standardized manner at the beginning of the pregnancy and this may certify the reliability of data.

Strength points may include the multicenter design of the protocol, the large sample involved, and the standardized, validated, and reliable measurements employed. Also, the local protocol supported the referral of women reporting a higher risk of PD at T1 to the following specific services: ambulatories of psychiatry and psychotherapy at Policlinico di Foggia (delivering individual psychotherapy with a cognitive-behavioral approach) and local mental health centers in Foggia, when preferred.

### Clinical implications

Our findings suggest a number of factors to be considered in the screening of depression in the perinatal period. The clinical monitoring of BMI variations during pregnancy is relevant for the prevention of physical complications as well as for detecting women’s emotional or affective symptoms in the follow-up. We argue that physical and psychological well-being are both connected and personality traits, for example, neuroticism, may be identified as vulnerability factors for both physical and psychological negative outcomes among pregnant women. Thus, anthropometric and psychological characteristics should be routinely assessed for the screening of pregnancy complications and PD.

### Conclusions

Here we conclude that overweight and obesity may be considered among the risk factors for PD and complications during pregnancy. Some other characteristics such as personality traits of neuroticism and higher physical comorbidity seem to lead to additional vulnerability in terms of higher weight gain and poor depressive and pregnancy outcomes. Also, lower levels of education may significantly impact on the risk of depression and higher gestational weight gain in pregnancy. These findings might also suggest that a preventive psycho-educational program, beyond women’s educational background and personality traits, may improve the management of physical and emotional issues, and may reduce the risk of PD and complications during pregnancy.

## Data Availability

The ethics committee did not grant permission to share study data with third parties or to upload data in anonymized form.
